# Cu^II^(atsm) Attenuates Neuroinflammation

**DOI:** 10.3389/fnins.2018.00668

**Published:** 2018-09-24

**Authors:** Xin Yi Choo, Jeffrey R. Liddell, Mikko T. Huuskonen, Alexandra Grubman, Diane Moujalled, Jessica Roberts, Kai Kysenius, Lauren Patten, Hazel Quek, Lotta E. Oikari, Clare Duncan, Simon A. James, Lachlan E. McInnes, David J. Hayne, Paul S. Donnelly, Eveliina Pollari, Suvi Vähätalo, Katarína Lejavová, Mikko I. Kettunen, Tarja Malm, Jari Koistinaho, Anthony R. White, Katja M. Kanninen

**Affiliations:** ^1^Department of Pathology, The University of Melbourne, Melbourne, VIC, Australia; ^2^Department of Anatomy and Developmental Biology, Monash University, Melbourne, VIC, Australia; ^3^Department of Pharmacology and Therapeutics, The University of Melbourne, Melbourne, VIC, Australia; ^4^Florey Institute of Neuroscience and Mental Health, Parkville, VIC, Australia; ^5^A.I. Virtanen Institute for Molecular Sciences, University of Eastern Finland, Kuopio, Finland; ^6^Cell and Molecular Biology, QIMR Berghofer Medical Research Institute, Brisbane, QLD, Australia; ^7^Australian Synchrotron, Clayton, VIC, Australia; ^8^School of Chemistry, Bio21 Institute for Molecular Science and Biotechnology, The University of Melbourne, Melbourne, VIC, Australia; ^9^Neuroscience Center, University of Helsinki, Helsinki, Finland

**Keywords:** microglia, astrocyte, inflammation, neurodegeneration, copper

## Abstract

**Background:** Neuroinflammation and biometal dyshomeostasis are key pathological features of several neurodegenerative diseases, including Alzheimer’s disease (AD). Inflammation and biometals are linked at the molecular level through regulation of metal buffering proteins such as the metallothioneins. Even though the molecular connections between metals and inflammation have been demonstrated, little information exists on the effect of copper modulation on brain inflammation.

**Methods:** We demonstrate the immunomodulatory potential of the copper bis(thiosemicarbazone) complex Cu^II^(atsm) in an neuroinflammatory model *in vivo* and describe its anti-inflammatory effects on microglia and astrocytes *in vitro*.

**Results:** By using a sophisticated *in vivo* magnetic resonance imaging (MRI) approach, we report the efficacy of Cu^II^(atsm) in reducing acute cerebrovascular inflammation caused by peripheral administration of bacterial lipopolysaccharide (LPS). Cu^II^(atsm) also induced anti-inflammatory outcomes in primary microglia [significant reductions in nitric oxide (NO), monocyte chemoattractant protein 1 (MCP-1), and tumor necrosis factor (TNF)] and astrocytes [significantly reduced NO, MCP-1, and interleukin 6 (IL-6)] *in vitro*. These anti-inflammatory actions were associated with increased cellular copper levels and increased the neuroprotective protein metallothionein-1 (MT1) in microglia and astrocytes.

**Conclusion:** The beneficial effects of Cu^II^(atsm) on the neuroimmune system suggest copper complexes are potential therapeutics for the treatment of neuroinflammatory conditions.

## Introduction

Chronic neuroinflammation, manifested by increased activation of glial cells and excessive production of pro-inflammatory mediators, is one of the hallmark pathological features of several neurodegenerative diseases, including AD, multiple sclerosis, PD, and ALS. During neurodegeneration, chronic stimulation results in unresolved inflammation and creates a neurotoxic environment that exacerbates degeneration in the brain. The view that the brain is an immune-privileged site has been challenged by studies demonstrating the presence of bidirectional crosstalk between the CNS and the immune system (Louveau et al., 2015). It is now clear that during peripheral immune activation, the innate immune system signals to the brain, thereby inducing pro-inflammatory cytokine production and global gene expression changes in the brain (Thomson et al., 2014), as well as defects in spatial learning and memory (Jurgens et al., 2012; Eimerbrink et al., 2015; Elmore et al., 2015). Therapeutic approaches targeted to the modulation of both central and peripheral inflammation during neurodegeneration have shown beneficial outcomes in animal models and in human patients (Heneka et al., 2015; Venigalla et al., 2016).

Copper is required for many critical neuronal processes and functions, including differentiation, cell signaling, and synaptic transmission (D’Ambrosi and Rossi, 2015; Maureira et al., 2015; Hatori et al., 2016). Alterations to biometal homeostasis are implicated in several neurodegenerative diseases. For example, in PD, copper levels are diminished in the affected substantia nigra brain region (Genoud et al., 2017). In AD, X-ray fluorescence (XRF) analyses have shown that amyloid plaques are enriched for copper and zinc (Miller et al., 2006), yet several studies have demonstrated that many important regions such as the neocortex are in fact copper deficient in AD patient brains (Schrag et al., 2011; Rembach et al., 2013; Graham et al., 2014). Therefore, both the sequestration of copper in plaques and loss of copper-dependent functions have been proposed to contribute to AD pathology. Given the importance of biometal homeostasis on neuronal function, therapeutic targeting of abnormal biometal balance has been clinically demonstrated to be a potential therapeutic approach for neurodegeneration (Faux et al., 2010).

Modulating brain copper homeostasis is beneficial in animal models of neurodegenerative diseases. Copper bis(thiosemicarbazones) (btscs) are stable, lipophilic neutral copper(II) complexes that are capable of crossing cell membranes and the blood–brain barrier (Donnelly et al., 2008; Torres et al., 2016). Changes to the backbone of the ligand change the CuII/I reduction potential and lead to changes in the propensity of Cu-btscs to release Cu(I) inside cells (Donnelly et al., 2012). Metal delivery and metal redistribution with these complexes reduced disease-associated cognitive decline and improved learning and memory in AD model mice (Crouch et al., 2009), preserved motor function and survival in ALS mice (Soon et al., 2011; McAllum et al., 2013; Roberts et al., 2014), and improved both motor and cognitive functions in mouse models of PD (Hung et al., 2012). The copper bis(thiosemicarbazone) Cu^II^(atsm) is currently under clinical investigation in ALS and PD patients (NCT02870634, NCT03136809, and NCT03204929). In addition, it has been shown that other copper modulating compounds also improve learning and memory in AD model mice (Malm et al., 2007; Adlard et al., 2011), while dietary treatment of AD model mice with copper (Bayer et al., 2003), and crossing AD model mice with ATP7b mutant mice (Phinney et al., 2003), which display elevated copper levels, both result in extended lifetime and reduced production of amyloid beta (Aβ). The ability of the metal complexes Cu^II^(gtsm) and Cu^II^(atsm) to improve cognition in AD and motor performance in ALS has been attributed to reduction of Aβ oligomers and tau phosphorylation (Crouch et al., 2009), as well as the ability to improve the copper content of the metallo-protein Cu/Zn superoxide dismutase (SOD1) (Roberts et al., 2014) and reduce nitrosative damage (Soon et al., 2011). However, the observed reductions in the activation status of microglia and astrocytes in ALS and stroke mice treated with the copper complex Cu^II^(atsm) (Soon et al., 2011; Roberts et al., 2014; Huuskonen et al., 2017) suggest a potential anti-inflammatory effect of copper delivery *in vivo*, that has not been fully explored.

Inflammation regulates the expression of critical metal ion transport proteins in the brain (Terwel et al., 2011; Pelizzoni et al., 2013; Raha et al., 2013; Urrutia et al., 2013; Zhang et al., 2014; Michaelis et al., 2015), and several inflammatory proteins and transcription factors are dependent on metal ions for their activity. Inflammation also regulates metal buffering proteins such as the metallothioneins (Liu et al., 2014; Zhou et al., 2014; Luo et al., 2015). Metallothioneins are present in elevated levels in neurodegenerative diseases including AD and PD (Duguid et al., 1989; Adlard et al., 1998; Carrasco et al., 2006; Hidalgo et al., 2006; Michael et al., 2011) influence inflammatory responses in neurodegenerative diseases (Potter et al., 2007; Chung et al., 2009; Kim et al., 2012), and their deficiency is reported to enhance inflammatory cell responses (Potter et al., 2007). Even though the molecular links between metals and inflammation have been demonstrated, little information exists on the effect of copper modulation on brain inflammation.

In this study, we investigated the immunomodulatory potential of the neuroprotective copper agent Cu^II^(atsm) in a neuroinflammatory model *in vivo* and examined the effects of the copper complex on microglia and astrocytes *in vitro*. We demonstrate that Cu^II^(atsm) reduces brain inflammation caused by peripheral administration of bacterial LPS. We demonstrate that Cu^II^(atsm) has cell-specific inflammation-reducing actions *in vitro*, and report that its anti-inflammatory actions are associated with increased expression of *Mt1*, encoding the neuroprotective protein metallothionein-1 (MT1). The observed anti-inflammatory actions could account for some of the previously observed neurotherapeutic effects of copper delivery.

## Materials and Methods

### Animals

Four-month-old C57BL/6J male mice (*n* = 5–6/group) were used in this study. Male mice were used, because previous studies suggest that brain copper levels depend more on strain and age rather than sex (Maynard et al., 2002). Animals were housed in normal conditions with 12-h light/dark cycle and water and food available *ad libitum*. Animal handling and experimentation were performed in accordance with the national regulation of the usage and welfare of laboratory animals and approved by the Animal Experiment Committee in State Provincial Office of Southern Finland.

### Treatment Trials

Lipopolysaccharide from *Escherichia coli* (clone 055:B5, Sigma-Aldrich, St. Louis, MO, United States) was dissolved to 1 mg/ml in sterile PBS and intraperitoneally administered to 4-month-old mice at a dose of 1 mg/kg. Two hours after LPS administration, mice were treated with 60 mg/kg Cu^II^(atsm) dissolved in SSV solution containing 0.9% (w/v) NaCl, 0.5% (w/v) sodium carboxymethylcellulose, 0.5% (v/v) benzyl alcohol, and 0.4% (v/v) Tween 80, or SSV alone by oral gavage. Cu^II^(atsm) was prepared according to published procedures (Gingras et al., 1962). 23.5 h after LPS administration, mice were injected with MPIO-VCAM-1 (conjugate) that was prepared according to Montagne et al. (2012) by coating MyOne Tosyl Activated Dynabeads (Thermo Fisher Scientific, Waltham, MA, United States) with 0.1 M sodium borate buffer (pH 9.5) and conjugating with VCAM-1 antibody (40 μg/1 mg of beads, BD Biosciences, San Jose, CA, United States) in 3 M ammonium sulfate at 37°C for 48 h . The conjugate was blocked with 0.5% (w/v) bovine serum albumin (BSA) in PBS-Tween for 24 h at 37°C and stored in 0.1% (w/v) BSA in PBS-Tween at 4°C. Each animal received an intravenous dose of the MPIO-VCAM-1 conjugate containing 1.0 mg/kg of Fe 30 min prior to imaging.

### Imaging of Brain Inflammation *in vivo*

Magnetic resonance imaging was performed using a Biospec 9.4 T/12 cm system (Bruker, Germany) using a volume coil transmit/surface coil receive pair (Rapid Biomed, Germany) to produce 3D T2^∗^ weighted images (spatial resolution of 78 μm × 78 μm × 78 μm, TE/TR 12 ms/200 ms, flip angle of 25°, acquisition time 52 min). Four consecutive slices were used to construct minimum intensity projections. Systematic variation in signal intensity was removed prior to analysis by normalization to overall brain signal intensity. This approach allowed reliable detection of hypointense regions due to contrast agent. Data are the mean percentages of VCAM-1 signal area in nine consecutive minimum intensity projections per animal relative to control, beginning from the first slice where the hippocampus is visible. The VCAM-1 signal area was determined in oval regions of interest encompassing the coronal brain section within each image, and images were manually thresholded by a blinded investigator.

### Neonatal Astrocyte Cultures

For neonatal astrocyte-enriched cultures, newborn C57Bl/6J mice were decapitated, and the brains removed and placed into ice-cold preparation buffer (containing 68 mM NaCl, 2.7 mM KCl, 110 μM KH_2_PO_4_, 84.5 μM Na_2_HPO_4_, 29 mM sucrose, 2.8 mM glucose, 20 U/mL penicillin, and 34.4 pM streptomycin). Brains were sequentially passed through 250 and 135 μm gauze and centrifuged at 500 ×*g* for 5 min. Cell pellets were resuspended in growth medium consisting of high glucose DMEM (Life Technologies, Thermo Fischer Scientific) containing 10% (v/v) FBS (Bovogen, VIC, Australia), 20 U/mL penicillin, and 34.4 pM streptomycin (Life Technologies) and plated at a density of 150,000 cells/cm^2^. Cells were maintained at 37°C with 10% CO_2_. The growth medium was replaced every 7 days, and experiments were performed after 14 DIV.

### Adult Astrocyte Cultures

Astrocyte cultures were prepared as described previously (Pihlaja et al., 2011) with some modifications. Briefly, cortices were isolated from 6- to 8-week-old mice, the tissue suspended in Hank’s Balanced Salt Solution (Lonza, Allendale, NJ, United States) and centrifuged at 500 ×*g* for 5 min. After the addition of 0.25% trypsin-EDTA (Life Technologies, Great Island, NY, United States), the suspension was incubated at 37°C for 30 min with occasional shaking. After the addition of fresh culture medium to neutralize the effect of trypsin, the suspension was centrifuged at 500 ×*g* for 5 min. The cell suspension was added on top of Percoll (Sigma) and centrifuged. The layer of glial cells was washed once with fresh culture medium. The cells were plated onto poly-L-lysine pre-coated flask in DMEM/F12 media containing 10% FBS, 2 mM L-glutamine, 100 U/ml penicillin–streptomycin, and G5 supplement (Life Technologies, Great Island, NY, United States). Microglia were removed by shaking the plates before the experiments. Cells were used for experiments at passages 4–8.

### Primary Microglia

Microglia were harvested from astrocyte-enriched cultures plated at 65,000 cells/cm^2^ in six-well plates or T175 flasks after 18 DIV. Adherent glial cells were washed in PBS and incubated at 37°C with 1:5 dilution of 10× Trypsin-EDTA (Sigma-Aldrich) in high glucose DMEM until astrocyte detachment was complete (∼25 min). Astrocyte-conditioned media was added to adherent microglia in 6 well plates. Microglia in T175 flasks were scraped, counted and re-plated at a density of 65,000 cells/cm^2^. The media was changed to IMDM phenol red free media (Life Technologies) containing 10% (v/v) FBS, 20 U/mL penicillin, 34.4 pM streptomycin, and 2 mM L-glutamine (Life Technologies) the following day and cells were allowed to rest for a further 2 days prior to experiments.

### Cell Stimulation and Treatment

Cells were stimulated with 15 ng/ml IFNγ and 10 ng/ml TNFα (Peprotech, Stockholm, Sweden), or 20 μg/ml LPS (Sigma-Aldrich) for 24–48 h, as indicated. Cu^II^(atsm) (10 mM), prepared fresh in DMSO, was diluted in cell culture media and added to cells at concentrations from 0.2 to 2 μM for the indicated times. Minocycline (Sigma Aldrich) was used at 20 μM. Control groups were treated with the vehicle solutions of the treatment groups.

### MTT Assay

Cell viability was determined using the 3-(4,5-dimethylthiazol-2-yl)-2,5-diphenyltetrazolium bromide (MTT) assay (Amresco Inc., OH, United States). The MTT assay was performed by addition of 0.5 mg/ml (final concentration) MTT to the cell media. Cells were incubated at 37°C until blue crystals were visible by eye (approximately 30–60 min). The media was removed and replaced with 100 μl of DMSO per well to dissolve the crystals. One hundred microliter aliquots were distributed to 96-well plate wells and the absorbance at 585 nm was determined using an EnSpire^®^ Multimode Plate Reader (Perkin Elmer, Waltham, MA, United States). MTT reduction was blank corrected and expressed as a percentage of untreated controls.

### Lactate Dehydrogenase (LDH) Assay

Cell death was determined by measuring the enzymatic activity of extracellular LDH released from cells due to loss of membrane integrity indicating cell death using a Cytotoxicity Detection Kit (Roche, Basel, Switzerland) according to the manufacturer’s instructions. Briefly, media samples were combined with reaction mixture and absorbance measured at 492 nm using an EnSpire^®^ Multimode Plate Reader. Extracellular LDH activity was expressed as percentage of total cellular LDH activity determined from untreated cells lysed in culture media containing 1% triton X-100.

### Nitric Oxide (NO) Measurement

Nitric oxide concentrations in media were determined using a Nitric Oxide Assay Kit (Abcam, England, United Kingdom) or a Griess Reagent Kit (Thermo Fisher Scientific) according to the manufacturer’s protocols. Briefly, standards were prepared by serial dilution of the nitrite standard solution to final concentrations between 0 and 30 μM. 100 or 240 μl, for each respective kit, of freshly collected samples and standards (in duplicate) in 96-well microplate (Greiner Bio-One, Frickenhausen, Germany) were reacted with Griess reagents 1 and 2 (50 or 30 μl of each reagent, for each respective kit). Following 15 min of color development at RT, absorbance at 540 nm was determined using an EnSpire^®^ Multimode Plate Reader. Standard curves were constructed, and sample nitrite concentrations were calculated from standard curves. Results were expressed as percentage NO released relative to TNFα- and IFNγ-stimulated positive controls.

### Quantitative Real-Time Polymerase Chain Reaction

Total RNA was isolated from cells using the MagMax^TM^ Total RNA isolation kit or from frozen tissue samples using TRIzol^®^ (both from Invitrogen, Thermo Fisher Scientific), according to the manufacturer’s instructions. RNA concentrations were measured using the Qubit^®^ 2.0 fluorometer (Life Technologies) or NanoDrop Spectrophotometer (Thermo Fisher Scientific). RNA (200 ng–2 μg) was reverse transcribed using random hexamers and High Capacity cDNA kit (Life Technologies) or Maxima reverse transcriptase (Fermentas, Thermo Fisher Scientific). The relative expression levels of mRNA were measured according to the manufacturer’s protocol by qRT-PCR (StepOnePlus; Life Technologies, LightCycler 480; Roche, Basel, Switzerland, or QuantStudio 6; Thermo Fisher Scientific) using TaqMan chemistry and specific assays-on-demand target mixes (Arg1 Mm00475988_m1, Tgfb1 Mm01178820_m1, Ccl2 Mm00441242_m1, Tnf Mm00443260_g1 or Mm00443258_m1, Mt1 Mm00496660_g1, Il1a Mm00439620_m1, Il1b Mm00434228_m1, Il6 Mm00446190_m1, Nos2 Mm00440502_m1, Ptgs2 Mm00478374_m1; Life Technologies). The expression levels were obtained by normalizing the target gene to the geometric mean of GAPDH and beta-actin (Mm99999915_g1 and Mm02619580_g1, respectively; Life Technologies), and presented as fold change in the expression using the 2^-ΔΔCt^ method, where Ct is the threshold cycle value.

### Enzyme-Linked Immunosorbent Assay (ELISA)

Monocyte chemoattractant protein 1 (MCP-1/CCL2) and interleukin 6 (IL-6) concentrations were quantified by ELISA using the Mouse CCL2/JE/MCP-1 DuoSet and Mouse IL-6 DuoSet (R&D systems, Minneapolis, MN, United States), respectively, according to the manufacturer’s instructions. Results were expressed as percentages relative to the LPS or TNF-α and IFNγ-stimulated positive controls.

### Inductively Coupled Plasma Mass Spectrometry (ICP-MS)

Inductively coupled plasma mass spectrometry analysis of metal levels was performed as reported previously (Grubman et al., 2014b). Briefly, cell pellets collected for metal analysis were digested overnight in concentrated nitric acid (Aristar, BDH, Kilsyth, VIC, Australia), after which samples were heated for 20 min at 90°C. The volume of each sample was reduced to approximately 40–50 μl then 1 ml of 1% (v/v) nitric acid diluent was added to the samples. Some samples were analyzed using laser ablation ICP-MS in micro-droplet format as described previously (Yang et al., 2005; Hare et al., 2016). Briefly, cell cultures and certified element copper standard (100014-1; Choice Analytical, Australia) were prepared in phospho-safe extraction buffer (Merck). One microliter of each sample was pipetted onto a glass slide and left to air-dry overnight. Droplet residues were ablated off the slide surface using laser ablation and analysed using Iolite software (Paul et al., 2015; Hare et al., 2017). Measurements were made using an Agilent 7700x series ICP-MS instrument or a NWR-213 laser ablation unit (Electro Scientific Industries, Portland, OR, United States) hyphenated to an Agilent 8800 ICP-QQQ-MS. The concentrations of copper were calculated as μg of metal per mg of protein based on the protein concentration of the sample, as determined by BCA protein assay (Thermo Fisher Scientific).

### X-ray Fluorescence Microscopy (XFM)

The distribution of copper was mapped at the XFM beamline (Paterson et al., 2011) at the Australian Synchrotron on cryofrozen freeze-dried cell culture samples grown on SiN X-ray transparent membranes, as described previously (Grubman et al., 2014a). An incident beam of 12.7 keV X-rays was used to induce K-shell ionization of copper, while providing adequate separation of the Rayleigh and Compton peaks from the elemental fluorescence of interest. The incident beam was focused to a ∼1.5 μm spot (full-width at half maximum) using a Kirkpatrick–Baez mirror pair and specimens were fly-scanned through X-ray focus. The resulting XRF was collected in event mode using the low latency, 384-channel Maia XRF detector (Ryan et al., 2014) situated in the backscatter geometry. Full XRF spectra were used to reconstruct copper maps of the specimen using a virtual pixel size of 0.8 μm (corresponding to the sample interval), giving a pixel transit time of 25 μs. Reference foils of Mn and Pt (Micromatter, Canada), were scanned in the same geometry at regular intervals throughout the measurement and used as references to establish elemental quantitation. Deconvolution of the Maia data was performed using GeoPIXE v6.6 software (Kirkham et al., 2011).

### Statistics

Results were analyzed by ANOVA followed by Holm–Sidak’s multiple comparison test using GraphPad Prism software. Statistical significance was assumed if *p* < 0.05. Data presented in graphs represent the experimental mean ± SEM of the indicated number of independent experiments, each derived from multiple technical replicates.

## Results

### The Copper Complex Cu^II^(atsm) Is Anti-inflammatory *in vivo*

**FIGURE 1:**
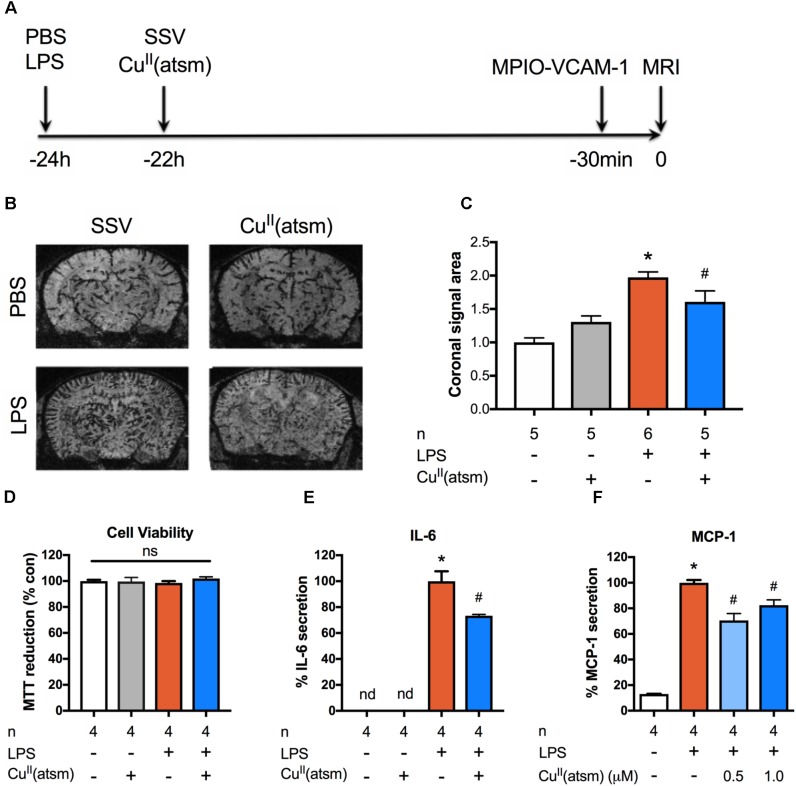
Copper delivery reduces inflammation induced by administration of LPS. **(A)** Timeline demonstrating time points for administration of the indicated compounds to adult mice and *in vivo* imaging of inflammation by MRI. **(B)** Representative MRI images acquired 30 min after intravenous administration of MPIO-VCAM-1 (24 h after LPS and 22 h after Cu^II^(atsm) or SSV) showing signal voids corresponding to MPIO binding. **(C)** Quantitative analysis of the volume of signal void in the brain induced by MPIO-VCAM-1 following treatment with LPS and Cu^II^(atsm). **(D–F)** Cultured adult astrocytes were stimulated with 20 μg/ml LPS with or without 0.5 μM Cu^II^(atsm) for 48 h and **(D)** MTT reduction, **(E)** IL-6 secretion, and **(F)** MCP-1 secretion were measured 48 h later. Sample sizes per group (*n*) as indicated. ^∗^*p* < 0.05 compared to control, ^#^*p* < 0.05 compared to LPS-treated group. ns, not significant. nd, not detected.

Peripheral inflammation induced by LPS triggers an inflammatory response that is characterized by increased expression of pro-inflammatory cytokines in the brain and plasma (Thomson et al., 2014). Furthermore, brain expression of VCAM-1, which plays a major role in the trafficking of leukocytes across the blood–brain barrier, is upregulated in response to systemic LPS (O’Sullivan et al., 2010; Thomson et al., 2014). To study the effect of a copper bis(thiosemicarbazone) complex on peripheral inflammation induced by systemic LPS, we employed a sophisticated *in vivo* MRI approach to measure brain inflammation that detects brain VCAM-1 expression in live animals. For this purpose, microsized particles of iron oxide targeting VCAM-1 (MPIO-VCAM-1) were intravenously administered to mice 30 min prior to imaging by MRI (**Figure 1A**). The MRI imaging revealed a robust, close to 100% increase in the expression of VCAM-1 at 24 h after administration of 1 mg/kg LPS (**Figures 1B,C**).

To investigate whether the copper complex, Cu^II^(atsm), possesses anti-inflammatory activity *in vivo*, mice were treated with a single dose of Cu^II^(atsm) by oral gavage after peripheral administration of LPS. As the inflammatory response in brain peaks 1 h after administration of LPS (Qin et al., 2007), mice were treated with Cu^II^(atsm) 2 h after LPS administration to investigate the effect of Cu^II^(atsm) on an ongoing inflammatory response. Mice were administered 60 mg/kg Cu^II^(atsm) as we have demonstrated this to be sufficient to induce neuroprotective effects *in vivo* (Huuskonen et al., 2017). MPIO-VCAM-1 imaging revealed a significant, ∼20% reduction in the level of brain VCAM-1 expression in the Cu^II^(atsm)-treated mice compared to control, SSV-treated mice at 24 h after LPS injection (**Figure 1C**). These data demonstrate the efficacy of the neuroprotective agent Cu^II^(atsm) in reducing acute cerebrovascular inflammation (measured as VCAM-1 expression) caused by systemic administration of LPS *in vivo*.

### Cu^II^(atsm) Alleviates LPS-Induced Inflammation in Primary Adult Astrocytes

Given that our *in vivo* findings during acute neuroinflammation suggest that copper may regulate inflammatory pathways, we next investigated inflammation *in vitro* utilizing adult astrocytes co-stimulated with LPS and Cu^II^(atsm) for 48 h. Neither 20 μg/ml LPS or 0.5 μM Cu^II^(atsm) alone or combined were toxic after 48 h (**Figure 1D**). This concentration of Cu^II^(atsm) is consistent with our previous pharmacokinetic studies which demonstrate Cu^II^(atsm) concentration reaches 0.6 μM in the mouse CNS following a single administration of 30 mg/kg (Hung et al., 2012). As expected, secretion of the cytokine IL-6 was strongly induced by LPS treatment in adult astrocytes (**Figure 1E**). This was significantly attenuated by co-treatment with Cu^II^(atsm). MCP-1 (CCL-2) is a chemoattractant cytokine, the suppression of which is known to be protective during neuroinflammation (Madrigal and Caso, 2014). To assess the effects of copper delivery on LPS-induced production of MCP-1, we next measured secreted MCP-1 (**Figure 1F**). LPS treatment induced a ∼10-fold increase in MCP-1 secretion from adult astrocytes, which was reduced by co-treatment with Cu^II^(atsm).

Although LPS is useful to investigate acute inflammatory insults, and cultured cells robustly respond to it, LPS is unlikely to directly stimulate brain cells *in vivo* due to poor blood–brain barrier penetration (Banks and Robinson, 2010). Therefore, we further studied the anti-inflammatory potential of Cu^II^(atsm) in cultured microglia and astrocytes stimulated with endogenous mediators of inflammation.

### Cu^II^(atsm) Exerts Anti-inflammatory Effects in Primary Microglia

**FIGURE 2:**
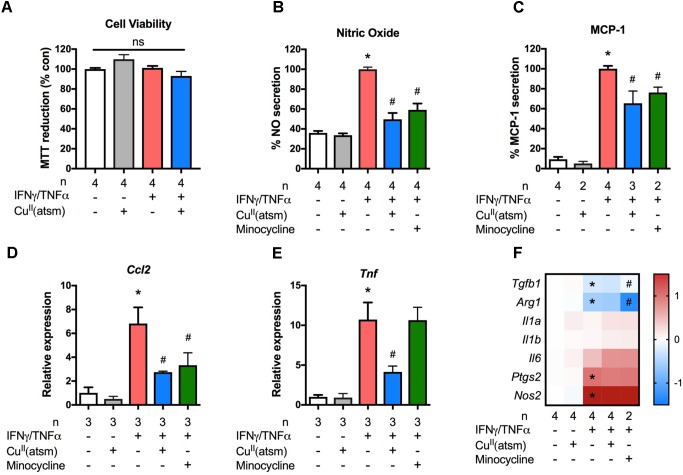
Copper delivery dampens microglial activation during inflammation. Primary microglia were stimulated with 15 ng/ml IFNγ and 10 ng/ml TNFα with or without 0.5 μM Cu^II^(atsm). **(A)** MTT reduction was measured to determine the cell viability 24 h later. Primary microglia were stimulated with 15 ng/ml IFNγ and 10 ng/ml TNFα with or without 0.5 μM Cu^II^(atsm) or 20 μM minocycline for 24 h. **(B)** Nitric oxide release into the media was measured 24 h later. **(C)** The secretion of MCP-1 into the media was measured by ELISA. qRT-PCR was used to measure microglial mRNA expression levels of **(D)** *Ccl2*, **(E)** *Tnf*, and **(F)** the indicated genes. **(F)** Presented as log fold-increase over control. Sample sizes per group (*n*) as indicated. ^∗^*p* < 0.05 compared to control, ^#^*p* < 0.05 compared to IFNγ/TNFα-treated group. ns, not significant.

Modulation of microglial activity is a viable therapeutic approach for diseases with a strong neuroinflammatory component. To determine whether copper bis(thiosemicarbazone) complexes modulate the inflammatory phenotype of microglia, we used a treatment paradigm where primary microglia were stimulated with the pro-inflammatory cytokines IFNγ and TNFα during co-treatment with Cu^II^(atsm). The maximum non-toxic doses of Cu^II^(atsm) for cultured microglia based on MTT and LDH assays were chosen for these experiments (**Supplementary Figure S1**). The inflammation-modulating activity of Cu^II^(atsm) was also compared to that of the well-known anti-inflammatory agent, minocycline (Malm et al., 2010). We first tested the effects of non-toxic concentrations (**Figure 2A**) of Cu^II^(atsm) on IFNγ/TNFα-induced NO production. The amount of NO was increased 2.5-fold after the 48 h IFNγ/TNFα stimulation (**Figure 2B**). Co-treatment with 0.5 μM Cu^II^(atsm) significantly suppressed the IFNγ/TNFα-induced NO production by ∼50%, whereas Cu^II^(atsm) alone did not affect NO secretion.

We next measured secreted MCP-1 (**Figure 2C**) and the mRNA levels of the MCP-1 gene, *Ccl2*, (**Figure 2D**) in primary microglial cultures. As expected, treatment with IFNγ/TNFα increased the secretion of MCP-1 into the media and the mRNA level of *Ccl2*. When compared to IFNγ/TNFα stimulation, co-treatment with Cu^II^(atsm) reduced the MCP-1 protein secreted into the media by 40% (**Figure 2C**). This reduction was comparable to that conferred by minocycline (**Figure 2C**). Similarily, Cu^II^(atsm) treatment was effective at reducing the mRNA expression of *Ccl2* (**Figure 2D**). Co-treatment with Cu^II^(atsm) also reduced the mRNA expression of *Tnf* encoding TNFα (**Figure 2E**).

Further analysis of inflammatory gene expression revealed these effects of Cu^II^(atsm) to be quite selective. In contrast to *Ccl2* and *Tnf*, Cu^II^(atsm) did not significantly alter the expression of *Il1a*, *Il1b*, *Il6*, *Ptgs2*, or *Nos2* (**Figure 2F**). The latter is intruiging, as this gene codes for inducible NO synthase, and would be expected to be decreased by Cu^II^(atsm) in light of the attenuated NO levels (**Figure 2B**). As expected, IFNγ/TNFα significantly decreased expression of two anti-inflammatory genes, *Tgfb1* and *Arg1* (**Figure 2F**). While Cu^II^(atsm) did not alter this response, minocycline increased expression of *Tgfb1* and further decreased expression of *Arg1*.

### Cu^II^(atsm) Dampens the Activation of Primary Astrocytes During Inflammation

**FIGURE 3:**
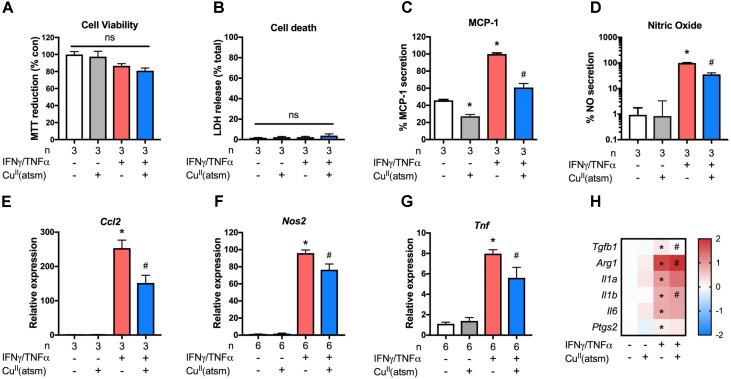
Anti-inflammatory effects of copper delivery in astrocytes. Neonatal astrocytes were stimulated for 48 h with 15 ng/ml IFNγ and 10 ng/ml TNFα with or without 2μM Cu^II^(atsm) for the final 24 h **(A–H)**. **(A)** MTT reduction and **(B)** LDH release was measured to determine the cell viability and cell death, respectively, after treatment. **(C)** MCP-1 secretion and **(D)** nitric oxide release were measured after treatment. qRT-PCR was used to measure astrocyte mRNA expression levels of **(E)** *Ccl2*, **(F)** *Nos2*, **(G)** *Tnf*, and **(H)** the indicated genes. **(H)** Presented as log fold-increase over control. Sample sizes per group (*n*) as indicated, or 6–7/treatment group for **(H)**. ^∗^*p* < 0.05 compared to control, ^#^*p* < 0.05 compared to IFNγ/TNFα-treated group. ns, not significant.

To determine whether the observed anti-inflammatory effects of copper delivery were specific for microglia only, we next employed a similar treatment paradigm for murine astrocytes with non-toxic concentrations (**Figures 3A,B**). In comparison to neonatal astrocytes stimulated with IFNγ/TNFα, co-treatment with Cu^II^(atsm) resulted in significantly reduced secretion of MCP-1 (**Figure 3C**) and NO (**Figure 3D**) into the culture media. This was accompanied by a corresponding attenuation of IFNγ/TNFα-induced *Ccl2* (**Figure 3E**), *Nos2* (**Figure 3F**), and *Tnf* (**Figure 3G**) expression by Cu^II^(atsm). Analysis of other inflammatory genes revealed IFNγ/TNFα increased expression of *Tgfb1* and *Arg1* in astrocytes, which was either attenuated or exacerbated by Cu^II^(atsm) co-treatment, respectively (**Figure 3H**). IFNγ/TNFα treatment increased expression of *Il1a*, *Il1b*, and *Il6*, but not *Ptgs2* (**Figure 3H**). Co-treatment with Cu^II^(atsm) did not diminish expression of these genes, and actually increased expression of *Il1a*.

### Cu^II^(atsm) Delivers Bioavailable Copper Into Cells

**FIGURE 4:**
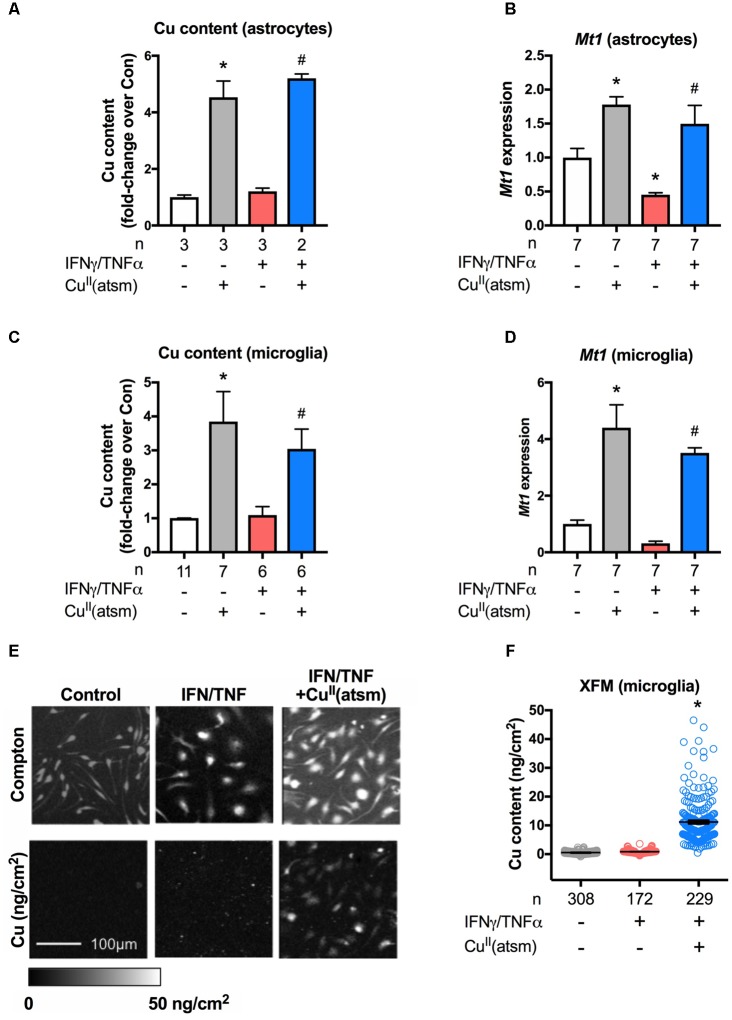
Cu^II^(atsm) delivers copper into cells. **(A,B)** Neonatal astrocytes were treated for 48 h with 15 ng/ml IFNγ and 10 ng/ml TNFα with or without 2 μM Cu^II^(atsm) for the final 24 h. **(C,D)** Microglia were treated for 24 h and with 15 ng/ml IFNγ and 10 ng/ml TNFα with or without 0.5 μM Cu^II^(atsm). Cellular copper content of **(A)** astrocytes and **(C)** microglia was measured by ICP-MS. mRNA expression of *Mt1* was measured by qRT-PCR in **(B)** astrocytes and **(D)** microglia. Sample sizes per group (*n*) as indicated. **(E)** Representative images of microglia analyzed for cellular copper content by XFM (reflected by elemental areal density). Scale bar 100 μm. **(F)** Quantification of XFM-determined copper levels per cell in microglia treated with 15 ng/ml IFNγ and 10 ng/ml TNFα with or without 0.5 μM Cu^II^(atsm). Cells per treatment group (*n*) as indicated. ^∗^*p* < 0.05 compared to control, ^#^*p* < 0.05 compared to IFNγ/TNFα-treated group.

Metal accumulation in response to treatment with Cu^II^(atsm) was examined to determine the effect of the compound on metal delivery in primary microglia and neonatal astrocytes under basal and inflammatory conditions. Treatment of either astrocytes or microglia with Cu^II^(atsm) caused a significant increase in cellular copper levels as measured by ICP-MS (**Figures 4A,C**). IFNγ/TNFα alone did neither alter copper levels as compared to basal levels nor alter copper content when co-treated with Cu^II^(atsm) relative to Cu^II^(atsm) alone.

Metallothionein-1 is a critical metal-binding protein that is involved in the regulation of metals and the modulation of anti-inflammatory and anti-oxidative pathways (Huh et al., 2007). To determine whether MT1 is associated with the anti-inflammatory effects induced by copper delivery, *Mt1* expression was investigated. In contrast to reports indicating that *Mt1* expression is increased by pro-inflammatory conditions (De et al., 1990; Manso et al., 2011; Everhardt Queen et al., 2016), here *Mt1* expression was decreased when either astrocytes or microglia were treated with IFNγ/TNFα alone (**Figure 4B**; **Supplementary Figure S2**). Treatment of astrocytes or microglia with Cu^II^(atsm) increased *Mt1* expression (**Figures 4B,D**). This expression was unchanged by co-treatment with IFNγ/TNFα As *Mt1* expression is well known to be strongly regulated by copper levels *via* metal regulatory transcription factor 1 (Juarez-Rebollar et al., 2017), these results indicate that Cu^II^(atsm) is delivering bioavailable copper into cultured glial cells. That the presence or absence of IFNγ/TNFα did not alter Cu^II^(atsm)-mediated copper content or *Mt1* expression of either astrocytes or microglia indicates that inflammatory conditions do not alter the accumulation of copper by glial cells, at least in culture.

Finally, XFM was used next to investigate the delivery and subcellular localisation of copper into primary microglia. Inelastic scatter (Compton) of incident photons was used to define the boundaries of cells in order to quantify metal levels for each individual cell (**Figure 4E**). We observed a dramatic increase in cellular copper in response to IFNγ/TNFα plus Cu^II^(atsm) treatment, with little overlap in copper content per cell as compared to cells treated in absence of Cu^II^(atsm) (**Figure 4F**). Copper appears to be diffusely localized throughout microglia, with no discernable nuclear exclusion or punctate localisation. In microglia treated with Cu^II^(atsm), copper levels per cell are somewhat heterogeneous, with some cells accumulating much more copper than others. Together, these results demonstrate that Cu^II^(atsm) efficiently delivers copper into cells under both basal and inflammatory conditions.

## Discussion

Modulation of copper and inflammation are both beneficial during neurodegeneration (Soon et al., 2011; Hung et al., 2012; Heneka et al., 2013; Roberts et al., 2014; Spangenberg and Green, 2016), yet the direct inflammation-modulating effects of copper compounds have remained relatively unexplored. Here we demonstrate, for the first time, that a bis(thiosemicarbazone) copper complex has anti-inflammatory effects in cultured microglia and astrocytes, and moderates inflammation in mice subjected to acute peripheral inflammation.

The role of copper in neuroinflammatory conditions, including AD, is not clear. Multiple studies have shown that increases in free copper levels impairs the clearance of Aβ and worsens behavioral outcome in animal models of AD (Kitazawa et al., 2009; Singh et al., 2013; Kitazawa et al., 2016). Copper triggers activation of cultured microglia, causing them to release the pro-inflammatory cytokine TNFα and NO into the culture media (Hu et al., 2014; Yu et al., 2015). Accordingly, sequestering systemic copper *via* D-penicillamine or tetrathiomolybdate reduces systemic oxidative stress in AD patients (Squitti et al., 2002), and inhibits acute LPS-induced peripheral inflammatory reactions *in vivo* (Wei et al., 2011). However, these chelator studies did not assess inflammation in the brain, where in contrast, intracellular copper deficiency elicits a neurodegenerative phenotype in mice and promotes the activation of microglia (Zucconi et al., 2007). Together, these studies suggest that too much or too little copper is detrimental and that intracellular, physiological copper concentrations are essential for the prevention of neuroinflammation. In line with this, neuroinflammatory conditions, including AD, have profoundly disturbed copper homeostasis. Copper-induced Aβ deposition occurs concomitantly with a reduction in the amount of intracellular, bioavailable copper, rendering the AD brain copper deficient (Schrag et al., 2011; Choo et al., 2013). In contrast to the inflammatory effects of free copper treatments, we find that the copper bis(thiosemicarbazone) complex applied in the current study moderates peripherally induced neuroinflammation *in vivo* and attenuates inflammation in cultured glial cells. This is in line with our previous findings that modulation of copper by cell permeable copper-delivering compounds is potently neuroprotective in various *in vivo* models of neurodegeneration (Crouch et al., 2009; Adlard et al., 2011; Soon et al., 2011; Hung et al., 2012; McAllum et al., 2013; Roberts et al., 2014), and that these protective effects are accompanied by modulation of acute and chronic neuroinflammation *in vivo* (Soon et al., 2011; Roberts et al., 2014; Huuskonen et al., 2017). The discrepancy between free copper and these complexes is likely due to the specific actions of these copper complexes, redistributing copper ions, and circumventing the endogenous copper trafficking machinery to restore copper homeostasis. The strong neuroprotective effects of these complexes demonstrates the potential therapeutic utility of our approach: Cu^II^(atsm) is currently under investigation in clinical trials for the treatment of PD and ALS (NCT02870634, NCT03136809, and NCT03204929).

We demonstrated by MRI imaging that an acute inflammatory insult induces a robust cerebrovascular inflammatory phenotype, which is dampened by treatment with Cu^II^(atsm). Our findings corroborate earlier reports whereby the expression of VCAM-1 in the brain is upregulated in response to systemic LPS (O’Sullivan et al., 2010; Thomson et al., 2014). VCAM-1 is an adhesion molecule overexpressed by the activated cerebral vasculature during inflammation and is considered a promising marker for molecular imaging of neuroinflammation in CNS disorders (Gauberti et al., 2014). For the first time, we report that peripheral LPS-induced brain VCAM-1 expression is modulated by a copper complex. It has been shown that superoxide dismutase 1 (SOD1/Cu-Zn SOD) plays an important role in scavenging the superoxide anions generated by inflammatory challenge and limiting VCAM-1-mediated propagation of the injury (Ishigami et al., 2011). Since Cu^II^(atsm) is known to increase SOD1 copper content in animal models of ALS (Roberts et al., 2014), we hypothesize that the antioxidant reactions raised by Cu^II^(atsm) may contribute to reducing VCAM-1 expression after systemic LPS challenge. Interestingly, administration of copper-bound anti-inflammatory agents significantly reduces endothelial expression of VCAM-1 after acute injury in rabbits (Puranik et al., 2016). By contrast, an *in vitro* study has shown that treatment of human aortic endothelial cells with cupric sulfate increases VCAM-1 expression, indicative of increased inflammation (Wei et al., 2014). These apparently conflicting results can be reconciled by the fact that the latter study did not assess the effect of copper under inflammatory conditions, used supra-physiological concentrations of copper, and used free copper as opposed to a membrane-permeable copper delivery agent, which as discussed above does not allow for tight control of metal delivery, or a direct comparison to be made to our data. While examination of the attenuation of inflammation by Cu^II^(atsm) *in vivo* in the current study is limited to VCAM-1 expression, our previous studies demonstrate that Cu^II^(atsm) robustly attenuates the neuroinflammation associated with acute and chronic neurodegenerative insults (Soon et al., 2011; Roberts et al., 2014; Huuskonen et al., 2017). Nevertheless, future studies will be useful to examine additional markers of inflammation both in the CNS and periphery to further elucidate the effects of Cu^II^(atsm) in acute models of inflammation.

Inflammation in the CNS is regulated by microglia and to a lesser extent astrocytes. Our *in vitro* data support the notion that copper delivery is beneficial in these glial cells through the demonstration that under inflammatory conditions, treatment with Cu^II^(atsm) reduced secretion of NO and IL-6, reduced the expression of the gene encoding the classical pro-inflammatory molecule TNFα, and reduced both the expression of the gene encoding MCP-1 and the secretion of the protein into the culture media. However, no changes were observed in the expression of several other inflammatory genes. This may indicate that the anti-inflammatory effects of Cu^II^(atsm) are somewhat selective. Interestingly, NO secretion by microglia is attenuated by Cu^II^(atsm), but not the elevated expression of *Nos2.* This suggests post-transcriptional modifications are responsible for decreased NO secretion. Indeed, a direct interact of copper with NO species has been reported for microglia (Rossi-George and Guo, 2016). Such interactions could also influence other inflammatory mediators, and this remains to be determined. An alternative option for lack of change in expression is that more wide-ranging anti-inflammatory effects were not able to be observed under the conditions utilized in this study but may occur under different conditions. In support of this, minocycline has been reported to attenuate the expression of many inflammatory genes, however several were examined here and were found to be unaltered by minocycline treatment, including *Nos2*, *Il6*, *Il1b*, and *Ptgs2* (Matsui et al., 2010; Piotrowska et al., 2017). That Cu^II^(atsm) consistently induced more robust anti-inflammatory effects than minocycline supports the strong anti-inflammatory action of Cu^II^(atsm).

Arginase-1 and TGB-β are strongly induced in microglia and macrophages during anti-inflammatory conditions and are often used as a marker of anti-inflammatory/alternatively activated cells (Jha et al., 2016). However, mounting evidence suggests that this bipolar classification of microglia is not reflective of the complexity of dynamic microglial transcriptional fingerprints in the physiological and pathological brain (Ransohoff, 2016; Keren-Shaul et al., 2017). Our results are consistent with this, with microglia exhibiting decreased expression of both *Arg1* and *Tgfb1* in response to IFNγ/TNFα-induced inflammation, whereas both are increased in response to the same stimuli in cultured astrocytes. It is important to note that our astrocyte cultures contain a substantial subpopulation of microglia. The phenotype of both microglia and astrocytes are strongly influenced by communication with neighboring cells. For example, MCP-1 released by astrocytes is known to activate microglia, cause their pro-inflammatory phenotype switch, and to enhance their migratory ability (He et al., 2016), and the presence of even a small number of microglia is capable of altering the phenotype of cultured astrocytes (Liddelow et al., 2017). Given this extensive crosstalk between microglia and astrocytes, this mixed glial culture perhaps represents a more physiological model than either isolated microglia or astrocytes. Regardless, with the exception of *Arg1* and *Tgfb1*, that similar anti-inflammatory results were obtained in both cultured astrocytes and microglia further strengthens the validity of these effects and the likelihood of translation *in vivo*.

How does Cu^II^(atsm) trigger the anti-inflammatory effects demonstrated herein? Cu^II^(atsm) can deliver bioavailable copper into the CNS (Crouch et al., 2009; Roberts et al., 2014). Conditions consistent with oxidative stress promote the dissociation of copper from Cu^II^(atsm) (Sirois et al., 2018). This produces a targeted effect proven to be beneficial in animal models of conditions such as stroke and ALS, where neuroinflammation is strongly related to increased oxidative stress (Roberts et al., 2014; Huuskonen et al., 2017). Copper-induced increases in microglial and astrocytic *Mt1* expression have been reported by others (Agullo et al., 1998; Bulcke and Dringen, 2015) and are now confirmed by the current report. This increase indicates copper dissociation from Cu^II^(atsm). In addition to the previously reported mechanisms of action of Cu^II^(atsm) connected to increased antioxidant scavenging (Soon et al., 2011; Hung et al., 2012; Roberts et al., 2014), we show here that MT1 may also mediate the protective effects of Cu^II^(atsm). Existing reports demonstrate that metallothioneins, metal-buffering proteins with numerous protective functions, modulate anti-inflammatory pathways (Inoue et al., 2009). *Mt1* expression is also upregulated in ALS patients and model mice (Sillevis Smitt et al., 1994), in AD patients and in mice modeling the disease, specifically in cells surrounding amyloid plaques (Carrasco et al., 2006; Kim et al., 2012) and in astrocytes (Kim et al., 2012). *In vitro* studies have demonstrated that in addition to preventing amyloid neurotoxicity directly, MT1 also suppresses Aβ-dependent microglial activation and reduces the neurotoxicity of Aβ-stimulated microglia (Kim et al., 2012). Furthermore, *in vivo* studies have reported that over-expression of MT1 modulates key phenotypes of AD model mice in a gender- and age-related manner (Kim et al., 2012), and extends lifespan of ALS model mice (Tokuda et al., 2014). These studies demonstrate the critical role of MT1 during neuroinflammatory conditions, which may be involved in the therapeutic action of Cu^II^(atsm). While the *in vitro* results suggest inflammation does not alter Cu^II^(atsm) or copper accumulation, our previous studies indicate the accumulation of copper from Cu^II^(atsm) is enhanced under disease conditions (Donnelly et al., 2012; Roberts et al., 2014). Hence, it remains to be determined whether the pharmacokinetics of Cu^II^(atsm) *in vivo* are altered under acute inflammatory conditions.

To have clinical utility, anti-inflammatory therapeutics must be able to inhibit ongoing inflammation. For the *in vivo* analyses in the current study, as 1 h is reportedly sufficient for LPS to generate a robust inflammatory response (Qin et al., 2007), the effects of Cu^II^(atsm) administered 2 h after LPS are likely to indicate the attenuation of ongoing inflammation, rather than preventing the onset of inflammation. Similarly, while inflammation was induced in microglia simultaneously with Cu^II^(atsm) treatment, primary astrocytes were treated both simultaneously and 24 h following induction of inflammation. Both paradigms elicited similar results demonstrating Cu^II^(atsm) attenuates prevailing inflammation *in vitro*. Furthermore, our previous studies in animal models of several neurodegenerative conditions also demonstrate that Cu^II^(atsm) elicits robust protective effects even when administered post-symptom onset (Hung et al., 2012; McAllum et al., 2013; Huuskonen et al., 2017). Thus Cu^II^(atsm) can ameliorate ongoing inflammation.

## Conclusion

Available treatment options for neuroinflammatory diseases are inadequate and those available have limited efficacy. Discovering novel approaches that modulate neuroinflammation are therefore of immense therapeutic interest for these disorders. Here, we demonstrated robust inflammation-reducing effects of a cell permeable copper complex in both *in vivo* and *in vitro* models of neuroinflammation. Given our newly reported insights into its neuroimmune role, this study demonstrates that copper delivery is a strong candidate for the development of therapies for neuroinflammatory conditions.

## Ethics Statement

Animal handling and experimentation were performed in accordance with the national regulation of the usage and welfare of laboratory animals and approved by the Animal Experiment Committee in State Provincial Office of Southern Finland and the University of Melbourne Animal Ethics Committee (ethics ID. 1312831).

## Author Contributions

XC, JL, AG, MH, DM, JR, KK, LP, HQ, LO, CD, SJ, LM, DH, EP, SV, KL, MK, and JR performed experimental work, data analysis, and contributed to manuscript preparation. XC, AG, MH, LM, PD, TM, JR, JK, AW, and KK developed the concept of the project and contributed to the manuscript preparation.

## Conflict of Interest Statement

Collaborative Medicinal Development LLC has licensed intellectual property related to this subject from The University of Melbourne, where the inventors include PD and AW. The remaining authors declare that the research was conducted in the absence of any commercial or financial relationships that could be construed as a potential conflict of interest.

## Supplementary Material

FIGURE S1 Toxicity analysis of Cu^II^(atsm) in primary microglial cultures. Primary microglia were treated with the indicated concentrations of Cu^II^(atsm) for 24 h. **(A)** MTT reduction and **(B)** LDH release were measured after 24 h. *N* = 2–5/group. ^∗^*p* < 0.05 compared to control.

FIGURE S2 Inflammation decreases metallothionein-1 expression in microglia. Microglia were treated for 24 h with or without 15 ng/ml IFNγ and 10 ng/ml TNFα. mRNA expression of *Mt1* was measured by qRT-PCR. *N* = 7/group. ^∗^*p* < 0.05 compared to control.

## Abbreviations

Aβ: amyloid beta
AD: Alzheimer’s disease
ALS: amyotrophic lateral sclerosis
BCA: bichinchoninic acid
CNS: central nervous system
Cu^II^(gtsm): glyoxalbis(*N*(4)-methyl-3-thiosemicarbazonato)copper(II)
Cu^II^(atsm): diacetylbis(*N*(4)-methyl-3-thiosemicarbazonato)copper(II)
DIV: days *in vitro*
DMSO: dimethyl sulfoxide
ELISA: enzyme-linked immunosorbent assay
FBS: fetal bovine serum
GAPDH: glyceraldehyde 3-phosphate dehydrogenase
ICP-MS: inductively coupled plasma mass spectrometry
IFNγ: interferon gamma
IL: interleukin
iNOS: inducible nitric oxide synthase
LPS: lipopolysaccharide
MCP-1/CCL2: monocyte chemoattractant protein 1
MPIO-VCAM-1: microparticles of iron conjugated to vascular cell adhesion molecule 1 antibody
MRI: magnetic resonance imaging
MT1: metallothionein-1
MTT: 3-(4,5-dimethylthiazol-2-yl)-2,5-diphenyltetrazolium bromide
NO: nitric oxide
Nrf2: nuclear factor erythroid 2-like 2
PBS: phosphate-buffered saline
PD: Parkinson’s disease
PDTC: pyrrolidine dithiocarbamate
PFA: paraformaldehyde
qRT-PCR: quantitative real-time polymerase chain reaction
RT: room temperature
SSV: standard suspension vehicle
TNFα: tumor necrosis factor α
XFM: X-ray fluorescence microscope
