# Supplementary figures and images for: CuII(atsm) Attenuates Neuroinflammation
Source: Front Neurosci. 2018 Sep 24;12:668. doi: 10.3389/fnins.2018.00668 (PMC6165894; doi:10.3389/fnins.2018.00668)

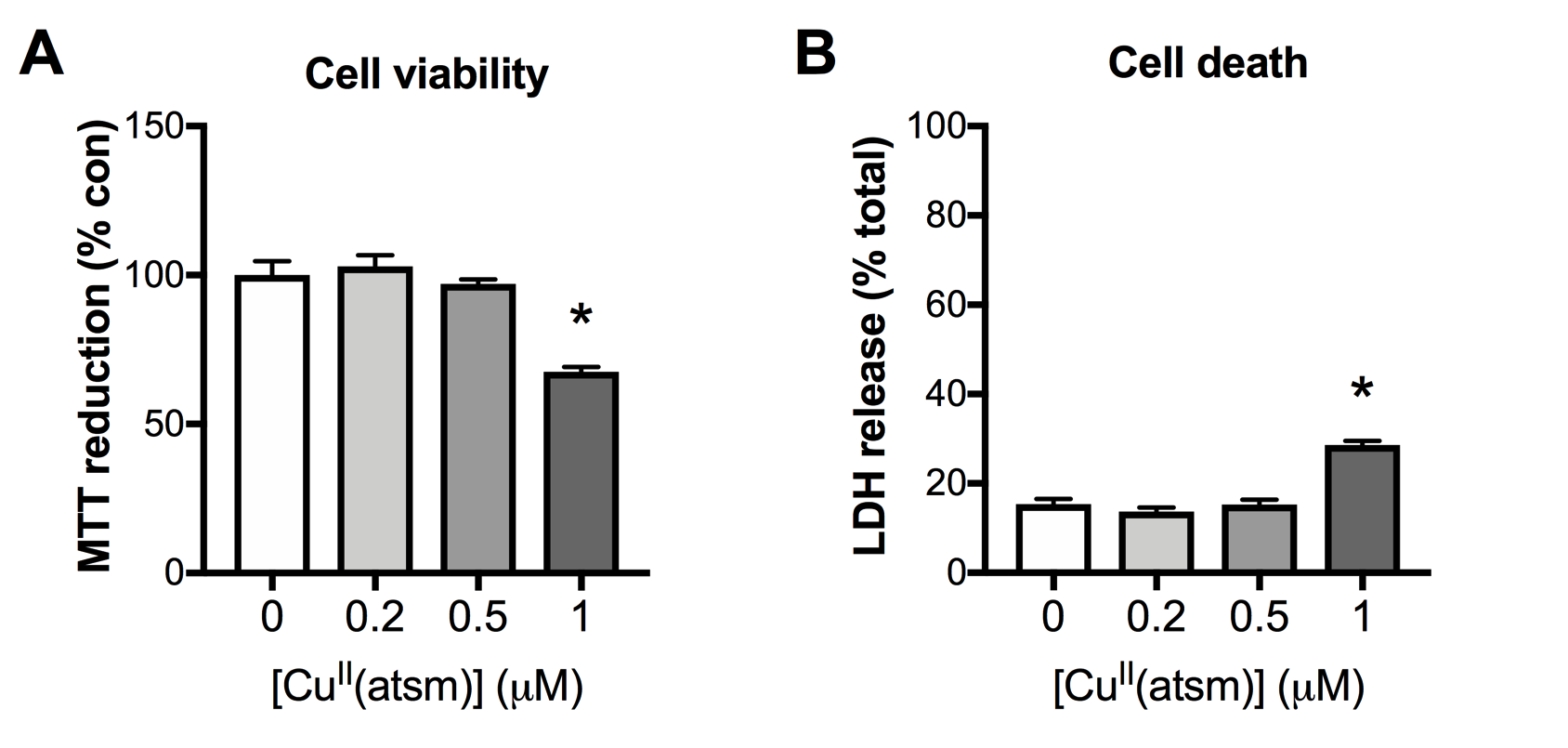

Supplement: FIGURE S1 — Toxicity analysis of CuII(atsm) in primary microglial cultures. Primary microglia were treated with the indicated concentrations of CuII(atsm) for 24 h. (A) MTT reduction and (B) LDH release were measured after 24 h. N = 2–5/group. ∗p < 0.05 compared to control. [file Image_1.tiff]

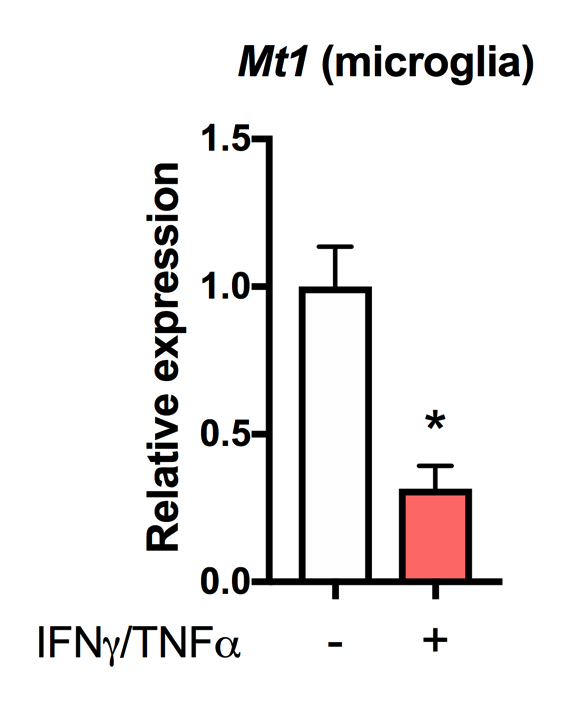

Supplement: FIGURE S2 — Inflammation decreases metallothionein-1 expression in microglia. Microglia were treated for 24 h with or without 15 ng/ml IFNγ and 10 ng/ml TNFα. mRNA expression of Mt1 was measured by qRT-PCR. N = 7/group. ∗p < 0.05 compared to control. [file Image_2.tiff]
